# CD49a Expression Identifies a Subset of Intrahepatic Macrophages in Humans

**DOI:** 10.3389/fimmu.2019.01247

**Published:** 2019-06-07

**Authors:** Glòria Martrus, Hanna Goebels, Annika E. Langeneckert, Janine Kah, Felix Flomm, Annerose E. Ziegler, Annika Niehrs, Sebastian M. Löbl, Kristina Russu, Leonard U. Hess, Wilhelm Salzberger, Tobias Poch, Björn Nashan, Christoph Schramm, Karl J. Oldhafer, Maura Dandri, Martina Koch, Sebastian Lunemann, Marcus Altfeld

**Affiliations:** ^1^Heinrich Pette Institute, Leibniz Institute for Experimental Virology, Hamburg, Germany; ^2^Internal Department of Medicine, University Medical Center Hamburg-Eppendorf, Hamburg, Germany; ^3^Center of Internal Medicine II, Brandenburg Medical School, University Hospital Brandenburg, Brandenburg, Germany; ^4^Martin Zeitz Center for Rare Diseases, University Medical Center Hamburg-Eppendorf, Hamburg, Germany; ^5^Department of Hepatobiliary and Transplant Surgery, University Medical Center Hamburg-Eppendorf, Hamburg, Germany; ^6^Clinic of Hepato-Pancreatico-Biliary Surgery and The Transplantation Center, First Affiliated Hospital, School of Life Sciences and Medical Center, University of Sciences & Technology of China, Hefei, China; ^7^Department of General & Abdominal Surgery, Asklepios Hospital Barmbek, Semmelweis University of Medicine, Hamburg, Germany; ^8^Department for General, Visceral and Transplant Surgery, University Hospital Mainz, Mainz, Germany

**Keywords:** monocytes/macrophages, human liver, tissue residency, cell activation, CD49a

## Abstract

Macrophages play central roles in inflammatory reactions and initiation of immune responses during infections. More than 80% of total tissue macrophages are described to be located in the liver as liver-resident macrophages, also named Kupffer cells (KCs). While studies in mice have established a central role of liver-resident KCs in regulating liver inflammation, their phenotype and function are not well-characterized in humans. Comparing paired human liver and peripheral blood samples, we observed significant differences in the distribution of macrophage (Mφ) subsets, with lower frequencies of CD14^hi^CD16^lo^ and higher frequencies of CD14^int−hi^CD16^int^ Mφ in human livers. Intrahepatic Mφ consisted of diverse subsets with differential expression of CD49a, a liver-residency marker previously described for human and mice NK cells, and VSIG4 and/or MARCO, two recently described human tissue Mφ markers. Furthermore, intrahepatic CD49a^+^ Mφ expressed significantly higher levels of maturation and activation markers, exhibited higher baseline levels of TNF-α, IL-12, and IL-10 production, but responded less to additional *in vitro* TLR stimulation. In contrast, intrahepatic CD49a^−^ Mφ were highly responsive to stimulation with TLR ligands, similar to what was observed for CD49a^−^ monocytes (MOs) in peripheral blood. Taken together, these studies identified populations of CD49a^+^, VSIG4^+^, and/or MARCO^+^ Mφ in human livers, and demonstrated that intrahepatic CD49a^+^ Mφ differed in phenotype and function from intrahepatic CD49a^−^ Mφ as well as from peripheral blood-derived monocytes.

## Introduction

Under homeostatic conditions, the liver represents an immune-privileged and tolerogenic organ, reflected by a general lack of immune responses induced by foreign antigens [with some exceptions, such as schistosomiasis (1)], and low rates of liver allotransplantation rejections (2, 3). The liver has however been suggested to play a central role in the regulation of host immune defenses (4, 5), as both gastrointestinal portal and systemic blood circulations reach the liver, exposing liver-resident immune cells to antigens (6). Livers contain a large population of non-parenchymal immune cells, including hepatic stellate cells (HSC), dendritic cells (DCs), macrophages (Mφ), Natural Killer (NK) cells and liver sinusoidal endothelial cells (LSEC) (7). Mφ and LSECs can absorb pathogens and molecules that entered the portal circulation after gastrointestinal translocation, as these cells are located within liver sinusoids, in close contact with the portal blood stream (8). Once peripheral blood monocytes migrate into tissues, they have been traditionally defined as Mφ, and serve as antigen presenting cells (APCs). In peripheral blood, MOs have been defined as CD14^hi^CD16^lo^ (classical), CD14^int−hi^CD16^int^ (intermediate), or CD14^lo^CD16^hi^ (non-classical) monocytes (9), with distinct functional capacities. In contrast, little is known about liver-derived human Mφ, and the markers that characterize these cells. Mφ in liver have been suggested to have dual origins, as they have the capacity to self-renew or to differentiate from recruited infiltrating peripheral blood monocytes (MOs) (10).

Liver-resident NK and T cells express CD49a, the alpha 1 subunit of α1β1 integrin, which retains cells in this organ by binding to two abundant molecules in the liver, collagen IV and laminin (11–15). Moreover, V-set and Ig domain-containing 4 (VSIG4), a molecule from the B7-related co-signaling family that binds to the complement component 3b (C3b and iC3b), was identified as a cellular marker for liver-derived Kupffer cells (KC) in mice, and was also suggested to be a marker for human macrophages residing in peritoneum and livers (16–22). Recently, an approach using RNA single cell sequencing of human liver tissues confirmed VSIG4 and identified the pattern recognition receptor (PRR) MARCO as specific markers of intrahepatic Mφ (ihMφ) (23). In mouse models, the presence of VSIG4^+^ KCs was critical in regulating responses of liver-resident T- and NKT-cells (24), suggesting a role of this molecule in limiting inflammatory tissue damage. MARCO^+^ cells were detected mainly in periportal areas of the liver and had lower responses to LPS/IFN-γ stimulation (23). However, little is known about the expression of CD49a, VSIG4, and MARCO on human liver-derived Mφ, and the functionality of these cells.

In this study, matched peripheral blood MOs and intrahepatic Mφ (ihMφ) were phenotypically and functionally characterized to identify specific markers expressed by these different subpopulations. Our studies identified a population of CD49a^+^, VSIG4^+^, and/or MARCO^+^ ihMφ in human livers. CD49a^+^ ihMφ exhibited cytokine responses at baseline and responded little to additional stimulation with TLR ligands. Altogether, our data suggest that CD49a serves as a marker to define intrahepatic Mφ and that CD49a^+^ ihMφ might play a role in regulating liver inflammation.

## Materials and Methods

### Study Cohort

Matched peripheral blood and liver tissue samples were obtained from individuals undergoing liver transplant surgery (Department of Hepatobiliary and Transplant Surgery, University Medical Center Hamburg-Eppendorf) or surgical tumor-free liver tissue resection due to liver metastases (Department of General and Visceral Surgery at the Asklepios Clinic Hamburg-Barmbek). Demographics of individuals included in this study for each experiment set are shown in Supplementary Tables 1–4. Liver samples from a total of 33 individuals were used to generate the data in this study. Studies were approved by the Institutional Review Board of the medical faculty at the University of Hamburg (PV4898, PV4081, PV4780, and WF-021/11). All study subjects provided written informed consent.

### Cell Preparation

Peripheral blood samples from each individual participating in this study were collected before or during surgery and processed within 2 h. Peripheral blood mononuclear cells (PBMCs) samples were prepared using Ficoll-Hypaque centrifugation (Biocoll), as described previously (25, 26). Collected liver tissue was stored on ice for a maximum of 30 min before processing and mechanically sliced into pieces of 0.5-1 cm^3^ under BSL3^**^ laboratory conditions (Biosafety Level 3^**^), as described (14, 27). If the amount of isolated liver cells obtained was higher than 1 × 10^9^ total cells, an additional purification step was performed. In brief, cells were diluted with PBS, centrifuged twice at 40xg for 4 min at room temperature (RT) and supernatant containing intrahepatic leukocytes (IHLs) was recovered and transferred to a new tube. PBS was added to obtain a final volume in the tube of 50 mL, cells were centrifuged at 400xg for 7 min and supernatant was discarded. This washing step was repeated twice. Cell pellets were resuspended in 4.5 mL PBS (final volume) and mixed with OptiPrep solution (Sigma-Aldrich) in a 15 mL falcon tube. 1 mL of PBS was carefully layered on top of the cell/Optiprep suspension and tubes were centrifuged for 20 min at 400xg without breaks. The interphase containing the erythrocyte/leukocyte populations was collected and cells were washed once with PBS. The cell pellet containing intrahepatic leukocytes (IHLs) was treated with ACK Lysis buffer (Sarsted AG&Co) following manufacturer's instructions and finally resuspended in RPMI+10% FBS for subsequent experiments. Enzymatic treatment was not used to avoid cleavage and degradation of cell surface receptors [data not shown and (28)].

### Phenotyping of Monocytes/ Mφ Populations Using Flow Cytometry

Freshly isolated IHLs and PBMCs (2 × 10^6^ cells) were used for flow cytometry phenotyping and the same voltages and settings were applied on paired samples. For the surface staining we used the antibodies summarized in Supplementary Table 5. Zombie aqua (Biolegend) was used to discriminate dead cells. Cells were analyzed using a BD LSR Fortessa and further analyses were performed with FlowJov10 software. The same gating strategy was applied for PBMCs and IHLs from the same donor in the analysis of monocytes/Mφ populations (Supplementary Figures 1A,B). t-SNE analyses were performed using the Cytobank (USA) platform. The corresponding isotype controls for VSIG4, CD49a and MARCO staining are shown in Supplementary Figure 2A and Supplementary Table 5. MARCO was not well-detectable in unstimulated freshly isolated samples using flow cytometry. To test for antibody specificity, freshly isolated PBMCs were polarized with recombinant human M-CSF (Peprotech) at 40 ng/mL for 5 days, followed by IL-10 stimulation (Peprotech) at 10 ng/mL for 48 h, as recommended by the vendor (Thermofisher). Cells were stained to define monocytes/macrophages and MARCO (Supplementary Figure 2B).

### Immunofluorescence

To visualize the expression of surface markers CD49a CD68, MARCO, and VSIG4 on Mφ, frozen human liver biopsies were cut in 5 μm sections. To control for tissue and cellular morphology, 12 μm slides were also prepared and stained with Hoechst, as described below. Tissue slides were fixed and permeabilized in acetone for 10 min at RT, washed three times with 1 × PBS, blocked for 30 min with 10% BSA in PBS and subsequently incubated overnight at 4°C with the following antibodies: CD49a (FITC; TS217; Biolegend, dilution 1:100), CD68 (AF 405 or PE; JO217; Santa Cruz; dilution 1:100), MARCO (unlabeled; polyclonal; Invitrogen; 1:100) and VSIG4 (APC or PE-Cy7; JAV4; eBioscience; dilution 1:100). When MARCO staining was performed, slides were washed three times with 0.1%BSA/1 × PBS for 5 min each and a secondary goat anti-rabbit antibody was used (AF633; Invitrogen; 1:400). Tissue sections that were not stained with CD68-AF405 antibody were stained with Hoechst for 2 min. Tissue slides were mounted with fluorescein mounting media (Dako, Glostrup, Denmark) and visualized by confocal laser scanning microscopy (Microscope Biorevo BZ-9000, Keyence, Japan) using the same settings for all recorded areas. Unstained samples were used to assess the autofluorescence background of tissue samples and used to set the sensitivity of detectors (Supplementary Figure 3A). Fiji (ImageJ) was used to analyze all data, using a macro (Fiji measurement analyzer) (Supplementary Code 1), aiming at first applying a positive mask on CD68^+^ signal (*n* = 561) and then calculating the signal presence and intensity on CD68^+^ signal for VSIG4, MARCO and CD49a. The specificity of the binary definition of signals and their intensities defined by the macro on Fiji showed a reliable gating strategy (Supplementary Figure 4).

### Intracellular Cytokine Staining

Functional responses of monocytes and macrophages to TLR4 or TLR7/8 ligands were assessed in cell cultures of PBMCs or OptiPrep-isolated IHLs stimulated with either LPS (100 ng/mL, Invivogen) or CL097 (1 μg/mL, Invivogen) in a medium containing BFA (5 μg/mL, Sigma Aldrich), as previously described (29). Unstimulated cells were used as baseline control. At 16 h post-stimulation, cells were prepared for intracellular cytokine staining following the manufacturer's instructions (FIX & PERM® Cell Fixation & Cell Permeabilization Kit, Thermo Fisher Scientific) and antibodies from Supplementary Table 5. Cells were washed and measured using a BD LSR Fortessa. Further analyses and gating strategies were performed with FlowJo v10 software.

### Statistical Analysis

Statistical analyses were performed using Prism v7.03 (GraphPad Software Inc.). Since the sample size for the individual comparisons was under *n* = 30, data was analyzed as non-normally distributed (non-parametric). Matched analyses were performed using Wilcoxon matched pair signed rank test for comparisons and adjusted for multiple comparisons (FDR) using the original FDR method of Benjamini and Hochberg (Q = 1%). When samples were not paired, Mann-Whitney tests were applied. When not otherwise stated, *p* values < 0.05 were considered as statistically significant and were depicted in the corresponding figures.

## Results

### CD49a Is Expressed by Intrahepatic Mφ

**Figure 1:**
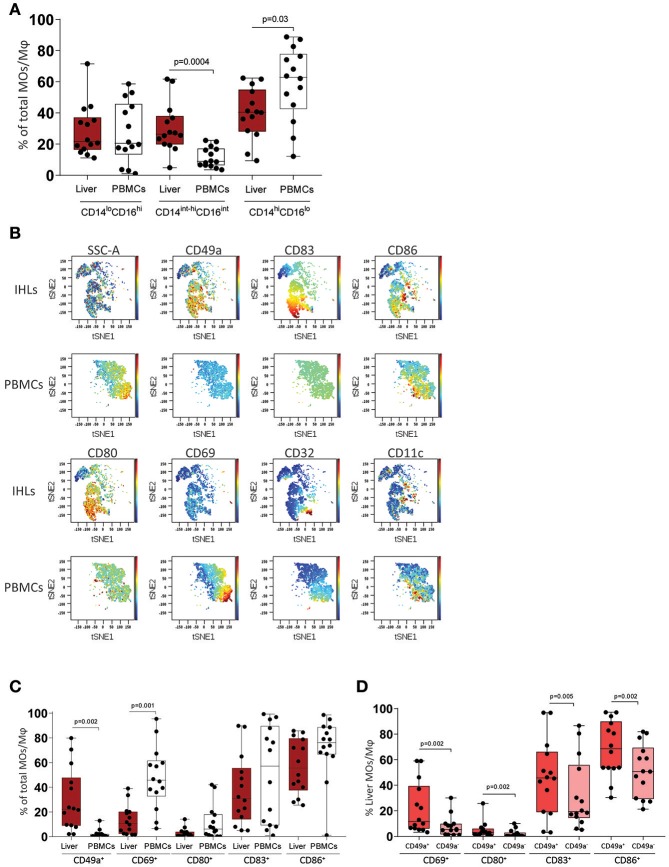
Immunophenotyping of macrophages (Mφ) and monocytes (MOs) in leukocytes from liver (IHLs) and blood (PBMCs). **(A)** Comparison of cell subset distribution between intrahepatic leukocytes (IHLs) (dark red) and matched peripheral blood mononuclear cells (PBMCs) (white) (*n* = 14). **(B)** t-SNE analysis of one representative sample showing the expression of CD49a, CD83, CD86, CD32, CD80, CD11c, and CD69 in both PBMCs and IHLs. **(C)** Scatter plots showing the frequency of CD49a^+^, CD69^+^, CD80^+^, CD83^+^, and CD86^+^ cells on Mφ on IHLs (dark red) and MOs on PBMCs (white). **(D)** Scatter plots showing the frequency of CD69^+^, CD80^+^, CD83^+^, and CD86^+^ cells on CD49a^+^ ihMφ (red) and CD49a^−^ ihMφ (salmon). Median with min-max range is shown (*n* = 14). All samples were analyzed using Mann-Whitney test and were additionally corrected for test-multiplicity using the original FDR method of Benjamini and Hochberg. Only statistically significant *p*-values are shown.

Monocytes and Mφ play a pivotal role in liver homeostasis and initiation of immune responses. We analyzed and compared the phenotypical profile of human peripheral blood monocytes (pbMOs) and intrahepatic Mφ (ihMφ) using flow cytometry. While no differences were observed in the CD14^lo^CD16^hi^ subset between liver and peripheral blood (Figure 1A), the proportion of CD14^int−hi^CD16^int^ cells was significantly upregulated and the proportion of CD14^hi^CD16^lo^ cells downregulated in liver-derived cells compared to peripheral blood (Figure 1A). In a representative t-SNE plot, monocytes and Mφ from PBMCs and liver diverged substantially, specially due to the differential expression of CD49a, CD80, CD83, CD69, and CD86 (Figure 1B, Supplementary Figures 1C,D).

The activation status of monocytes/Mφ was analyzed using CD69 (30), CD83 (31), CD80 (B7.1), and CD86 (B7.2) (32). Compared to pbMOs, ihMφ contained a lower proportion of cells expressing activation markers such as CD69, CD80, and CD86 (Figure 1C). IhMφ however contained almost exclusively a population of CD49a^+^ cells, which was very rare in pbMOs (Figure 1C), similarly to what has been described for intrahepatic NK cells (11, 14). No differences in the expression of the studied markers were observed between CD14^lo^CD16^hi^, CD14^int−hi^CD16^int^, and CD14^hi^CD16^lo^ monocytes/Mφ (data not shown). We subsequently gated on CD49a^+^ and CD49a^−^ ihMφ and compared the proportion of cells expressing activation markers. Compared to CD49a^−^ ihMφ, CD49a^+^ ihMφ contained higher proportion of cells expressing CD69, CD80, CD83, and CD86 (Figure 1D). Taken together, the data demonstrate that CD49a, but not CD69, can serve as a marker to identify intrahepatic Mφ, and that CD49a^+^ ihMφ represent the main Mφ population in livers expressing molecules of cellular activation.

### CD49a, VSIG4, and MARCO Serve as Markers for Intrahepatic Mφ

**Figure 2:**
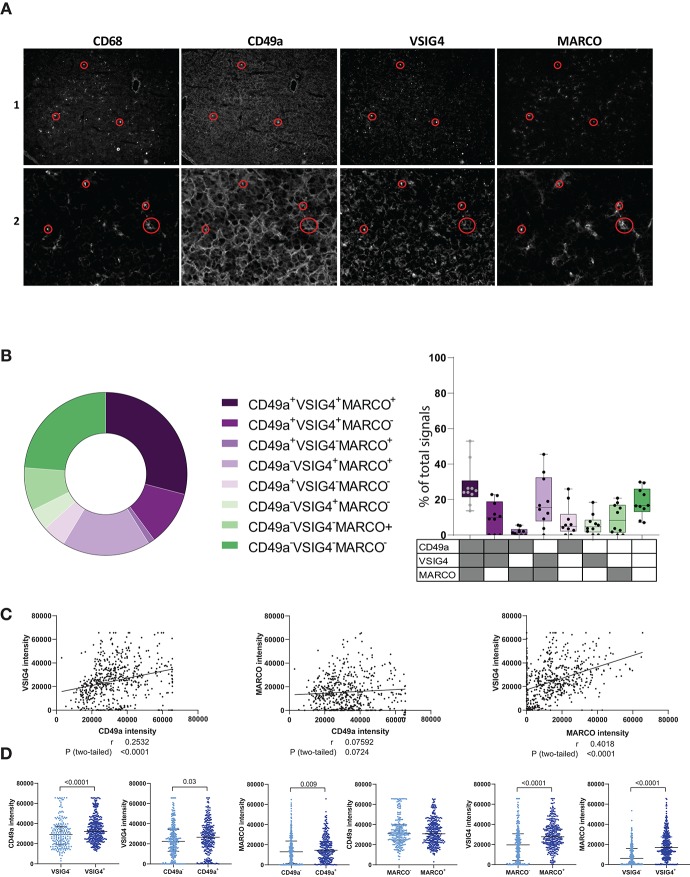
Immunofluorescence data showing CD49a, VSIG4 and MARCO expression in human intrahepatic macrophages. **(A)** Representative immunofluorescence staining of CD68, CD49a, VSIG4, and MARCO in frozen and acetone-fixed liver tissue sections in two liver samples. The red circles depict examples of co-expression of the four markers. **(B)** Boolean gating on masked CD68^+^ signals defining CD49a, VSIG4 and MARCO signal combinations (*n* = 561 signals from 10 tissue slides). **(C)** Correlation plots between CD49a and VSIG4 signal intensity (left), CD49a and MARCO (middle) and MARCO and VSIG4 (right) in cells defined as CD68^+^ (*n* = 561 pairs). **(D)** Scatter plots showing signal intensities of CD49a, MARCO and VSIG4 on CD68^+^ cells, categorized by VSIG4^+/−^ (*n* = 209 for VSIG4^−^ and *n* = 352 for VSIG4^+^), CD49a^+/−^ (*n* = 291 for CD49a^−^ and *n* = 270 for CD49a^+^), and MARCO^+/−^ signals (*n* = 247 for MARCO^−^ and *n* = 314 for MARCO^+^). Negative signals for each marker are represented in light blue and positive signals in dark blue. Median is depicted and error bars indicate the interquartile range, *p*-value determined by Mann–Whitney test.

To further characterize CD49a^+^ ihMφ in livers, CD68, VSIG4, and MARCO, three markers recently described to help define monocyte and macrophages in tissues (18, 23, 33), were used to stain CD49^+^ ihMφ by immunofluorescence of frozen acetone-fixed liver tissue slides. The staining confirmed that CD49a, MARCO and VSIG4 were expressed in liver tissues, as previously described in mice or humans (11, 14, 20, 23, 27), including on hepatocytes (Figure 2A, Supplementary Figures 3A,B). Liver tissues furthermore contained a high number of CD68^+^ cells, indicating high numbers of monocytes/Mφ (Figure 2A, Supplementary Figure 3). Specifically, our results showed that VSIG4^+^ Mφ in liver tissues, defined as positive for CD68, can also co-express CD49a and MARCO (Figures 2A,B). Boolean gating on the measured CD49a, VSIG4, and MARCO on CD68^+^ signals showed distinct populations of intrahepatic macrophages expressing different combinations of the three markers (Figure 2B). The three most prominent populations were either co-expressing all three markers, co-expressing VSIG4 and MARCO or none of the markers (Figure 2B). The presence of CD68^+^ signals only co-expressing MARCO and CD49a was scarce. Signal intensities of CD49a and VSIG4, as well as VSIG4 and MARCO, significantly correlated, indicating a strong co-expression pattern, while signal intensities of CD49a and MARCO did not correlate (Figure 2C). Furthermore, a binary analysis assessing the signal intensity of the three investigated markers (CD49a, MARCO and VSIG4) on populations either positive or negative for one of the other markers showed higher intensity for the respective markers on positive cells, with for example significantly higher CD49a intensity on VSIG4^+^ signals compared to VSIG4^−^ signals (Figure 2D). The only exception was CD49a intensity on MARCO^+/−^ signals (Figure 2D). Taken together, these data show the expression of CD49a, MARCO and VSIG4 on liver-derived Mφ populations, and identified Mφ populations expressing different combination of these markers.

**Figure 3:**
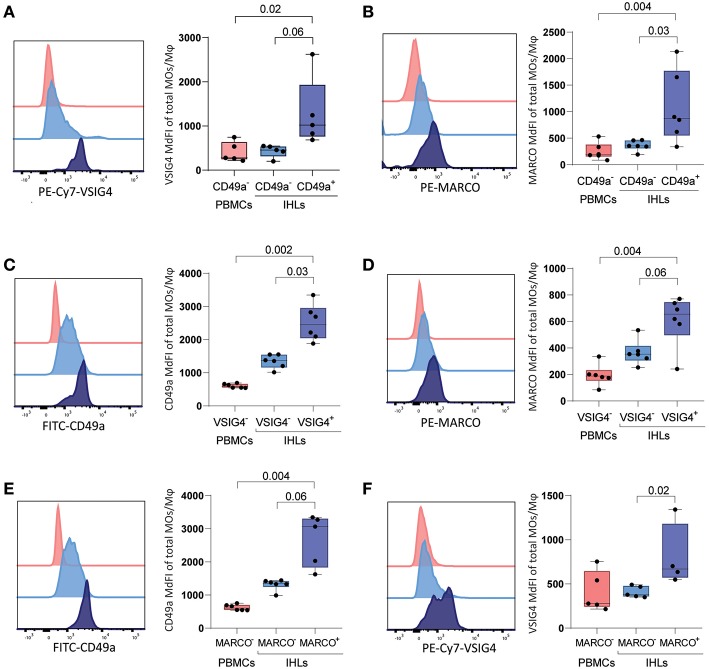
Expression of VSIG4, MARCO and CD49a on freshly isolated PBMCs and IHLs **(A)** (left) Representative histogram of VSIG4 expression on gated CD49a^−^ PBMCs (red), CD49a^−^ ihMφ (light blue), and CD49a^+^ ihMφ (dark blue). (right) Scatter plot summarizing VSIG4 MdFI on CD49a^−^ PBMCs (red), CD49a^−^ ihMφ (light blue) and CD49a^+^ ihMφ (dark blue) (*n* = 5). **(B)** (left) Representative histogram of MARCO expression on gated CD49a^−^ PBMCs (red), CD49a^−^ ihMφ (light blue) and CD49a^+^ ihMφ (dark blue). (right) Scatter plot summarizing MARCO MdFI on CD49a^−^ PBMCs (red), CD49a^−^ ihMφ (light blue) and CD49a^+^ ihMφ (dark blue) (*n* = 6). **(C)** (left) Representative histogram of CD49a expression on gated VSIG4^−^ PBMCs (red), VSIG4^−^ ihMφ (light blue) and VSIG4^+^ ihMφ (dark blue). (right) Scatter plot summarizing CD49a MdFI on VSIG4^−^ PBMCs (red), VSIG4^−^ ihMφ (light blue) and VSIG4^+^ ihMφ (dark blue) (*n* = 6). **(D)** (left) Representative histogram of MARCO expression on gated VSIG4^−^ PBMCs (red), VSIG4^−^ ihMφ (light blue) and VSIG4^+^ ihMφ (dark blue). (right) Scatter plot summarizing MARCO MdFI on VSIG4^−^ PBMCs (red), VSIG4^−^ ihMφ (light blue) and VSIG4^+^ ihMφ (dark blue) (*n* = 6). **(E)** (left) Representative histogram of CD49a expression on gated MARCO^−^ PBMCs (red), MARCO^−^ ihMφ (light blue) and MARCO^+^ ihMφ (dark blue). (right) Scatter plot summarizing CD49a MdFI on MARCO^−^ PBMCs (red), MARCO^−^ ihMφ (light blue) and MARCO^+^ ihMφ (dark blue) (*n* = 6 for PBMCs and MARCO^−^ IHLs, *n* = 5 for MARCO^+^ IHLs). **(F)** (left) Representative histogram of VSIG4 expression on gated MARCO^−^ PBMCs (red), MARCO^−^ ihMφ (light blue) and MARCO^+^ ihMφ (dark blue). (right) Scatter plot summarizing VSIG4 MdFI on MARCO^−^ PBMCs (red), MARCO^−^ ihMφ (light blue) and MARCO^+^ ihMφ (dark blue) (*n* = 5 for PBMCs and MARCO^−^ IHLs, *n* = 4 for MARCO^+^ IHLs). One sample was excluded due to the low proportion of MARCO^+^ cells. Median is depicted and error bars indicate the min-max range, *p*-value determined by Mann-Whitney test (PBMCs vs. IHLs) and Wilcoxon matched-pairs signed rank test when comparing IHL populations.

To confirm the immunofluorescence results, we subsequently stained matched unstimulated freshly-derived liver and PBMCs samples directly *ex vivo* for VSIG4, CD49a, and MARCO, and quantified marker expression using flow cytometry. CD49a^+^ ihMφ showed significantly higher median fluorescence intensities (MdFIs) of VSIG4- and also MARCO-expression compared to CD49a^−^ ihMφ or PBMCs (Figures 3A,B). CD49a and MARCO MdFIs were furthermore significantly higher on VSIG4^+^ ihMφ compared to VSIG4^−^ ihMφ (Figures 3C,D), and VSIG4 and CD49a MdFIs were higher on MARCO^+^ cells compared to MARCO^−^ cells (Figures 3E,F). These data acquired using multiparameter flow cytometry therefore largely mirrored data acquired by immunofluorescence analysis of liver samples. Overall, the data obtained with the two different techniques showed that CD49a, MARCO, and/or VSIG4 can be used as markers to identify intrahepatic Mφ.

### ihMφ Have a Higher Baseline Activation Status Compared to pbMOs

**Figure 4:**
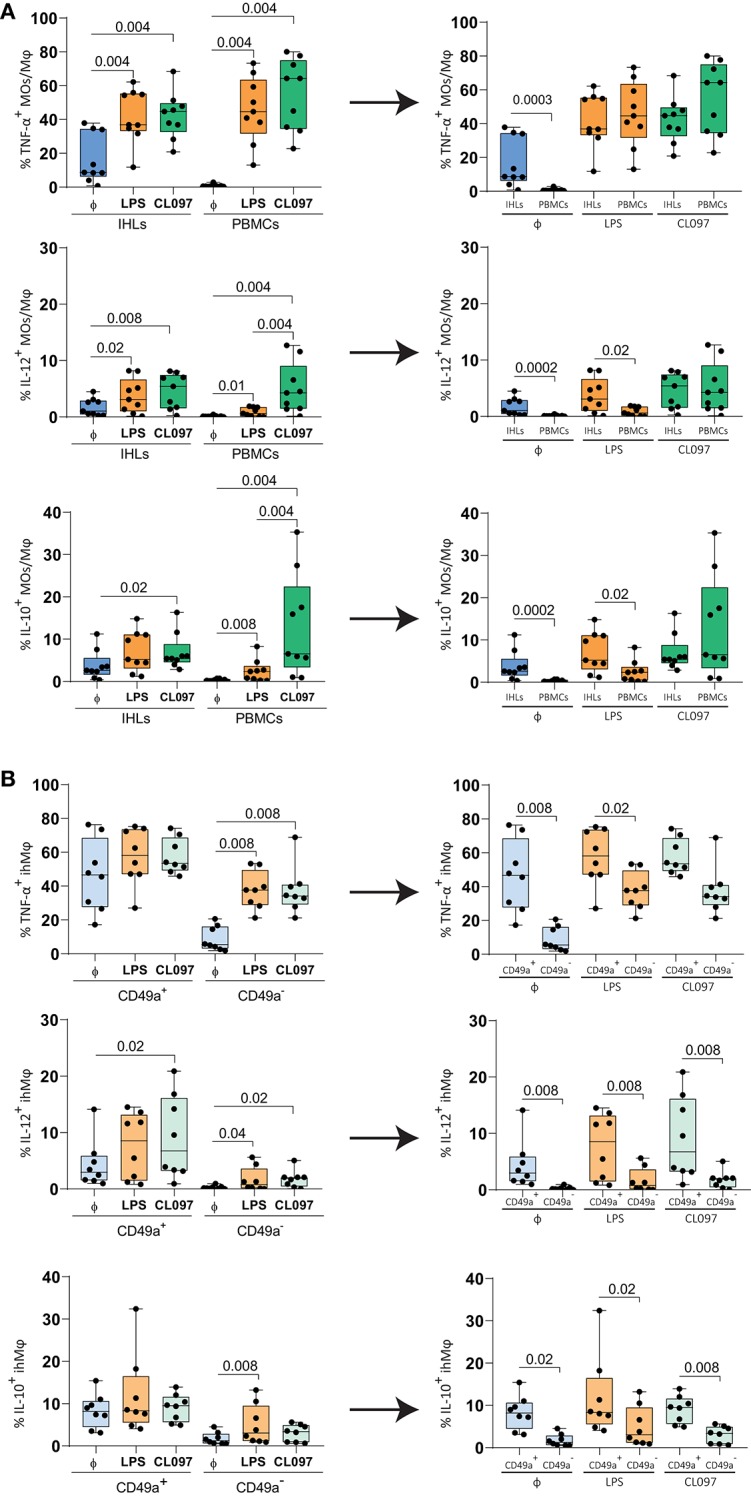
Functional responses of paired ihMφ and pbMOs to CL097 and LPS stimulations compared to unstimulated cells. **(A)** Scatter plots showing the comparison of the frequencies of TFN-α^+^ cells (top), IL-12^+^ cells (middle) and IL-10^+^ cells (bottom) between pbMOs and ihMϕ stimulated with LPS (orange) or CL097 (green), to unstimulated cells (blue) (*n* = 9). **(B)** Comparison of the frequency of TFN-α^+^ ihMφ (top) IL-12^+^ ihMφ (middle) and IL-10^+^ (bottom) cells between CD49a^+^ and CD49a^−^ ihMφ once stimulated with LPS (light orange) or CL097 (light green), to unstimulated cells (light blue) (*n* = 8). Medians and min-max range are showed. Liver and PBMCs comparisons were analyzed with a Mann–Whitney test and corrected for test-multiplicity using the original FDR method of Benjamini and Hochberg. Data in **(B)** was analyzed using a Wilcoxon matched-pairs signed rank test when comparing IHL populations. The arrows represent the reorganization of the same data. One sample was excluded from B due to the low amount of cells within the CD49a^+^ ihMφ gating. *Φ*: unstimulated. Only statistically significant *p*-values are shown.

To study the functional activity of ihMφ, we subsequently stimulated isolated IHLs and PBMCs with LPS (TLR4 stimulation) or CL097 (TLR7/8 stimulation), and used unstimulated cells to control for baseline activation. The proportion of cells expressing TNF-α and IL-12, pro-inflammatory cytokines secreted by MOs and Mφ, as well as IL-10, an anti-inflammatory cytokine, were quantified using flow cytometry. Compared to unstimulated cells, LPS- and CL097-stimulations significantly increased the proportion of TNF-α^+^, IL-12^+^, and IL-10^+^ cells (Figure 4A left panels). IhMφ responded to LPS and CL097 stimulation by increasing the proportion of TNF-α^+^ and IL-12^+^ cells when compared to unstimulated cells (Figure 4A left panel). No or little increase in IL-10^+^ ihMφ was observed after stimulation with LPS compared to baseline levels (Figure 4A, lower left panel). When comparing pbMOs and ihMφ, ihMφ included a significantly higher proportion of TNF-α^+^, IL-12^+^ and IL-10^+^ cells already at baseline, prior to stimulation (Figure 4A, right panel). CL097- and LPS-stimulations increased the proportion of TNF-α^+^ cells similarly in pbMOs and ihMφ (Figure 4A, right panel), and LPS-stimulation also increased the proportion of IL-12^+^ and IL-10^+^ ihMφ when compared to pbMOs (Figure 4A, right panel). Altogether, our data showed that ihMφ have a higher baseline production of cytokines, but are less reactive to further stimulation compared to pbMOs.

**Figure 5:**
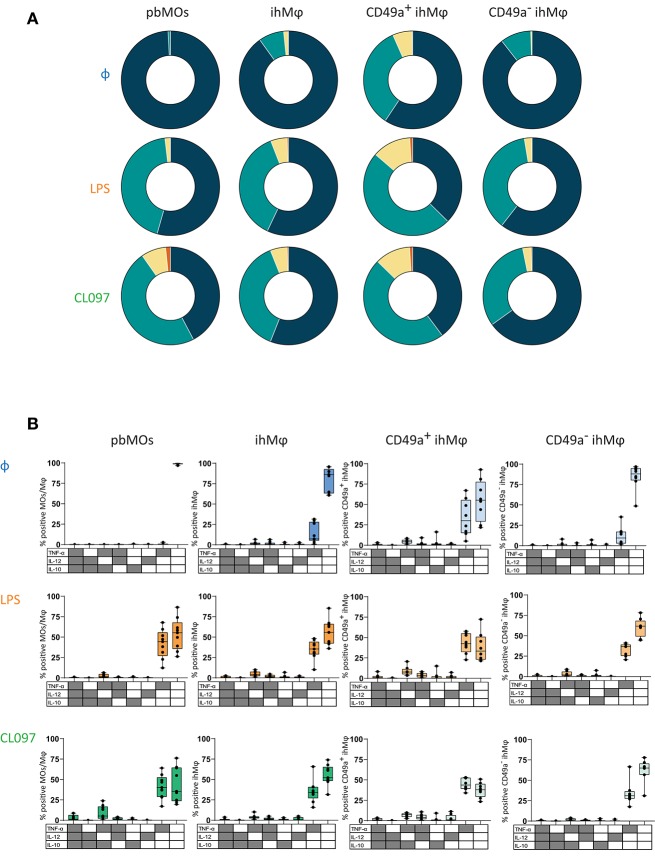
Polyfunction on cytokine responses of paired ihMφ and pbMOs. **(A)** Pie chart summarizing the percentage of MOs/Mφ expressing 0, 1, 2, or 3 cytokines, without stimulation or following LPS- or CL097-stimulation (blue: 0; green 1; yellow: 2; orange: 3) (*n* = 9 for pbMOs and ihMφ, *n* = 8 for CD49a^+^ ihMφ and CD49a^−^ ihMφ). **(B)** Boolean gating of cells expressing TNF-α, IL-12, and/or IL-10 on LPS-(orange), CL097-(green)stimulated pbMOs, ihMφ, CD49a^+^ ihMφ, and CD49a^−^ ihMφ. Unstimulated (blue) condition was used to calculate the baseline production of cytokines. One sample was excluded from the ihMφ analysis due to the low amount of cells within the CD49a^+^ ihMφ gating Medians and min-max range are showed.

We subsequently compared cytokine production between CD49a^+^ and CD49a^−^ ihMφ. Upon stimulation, CD49a^−^ ihMφ significantly increased the proportion of TNF-α^+^, IL-12^+^, and IL-10^+^ cells (the latter only with LPS stimulation) while the proportion of cytokine-positive cells was maintained at high levels within the CD49a^+^ ihMφ population (Figure 4B, left panel**)**. CD49a^+^ ihMφ represented the main Mφ population in livers already producing TNF-α, IL-12, and IL-10 at baseline without TLR-stimulation, and levels of cytokine production upon stimulation with LPS and CL097 were generally higher in CD49a^+^ ihMφ compared to CD49a^−^ ihMφ (Figure 4B, right panels). Finally, we assessed the polyfunctionality of all analyzed cell subsets based on IL-10, IL-12, and TNF-α production, including pbMOs, ihMφ and CD49a^+/−^ ihMφ using Boolean gating. Cells were stratified into 4 subsets depending on the number of cytokines produced (0–3). The number of cells producing one or more cytokines increased for all populations following stimulation with TLR ligands (Figure 5A). Following stimulation with LPS, ihMφ and in particular CD49a^+^ ihMφ included polyfunctional cells that produced 2 or more cytokines, while similar fractions of 2 or more cytokine-producing cells were observed across all cell subsets following CL097-stimulation (Figure 5A). A detailed analysis revealed that TNF-α was the main cytokine produced by unstimulated CD49a^+^ ihMφ, alone or in combination with IL-10 (Figure 5B). Upon LPS-stimulation, pbMOs and CD49a^−^ ihMφ had a similar cytokine pattern, while CD49a^+^ ihMφ contained higher proportion of TNF-α^+^IL-12^+^ cells (Figure 5B). In summary, CD49^+^ ihMφ exhibited higher baseline cytokine production compared to CD49a^−^ ihMφ cells, resulting in relatively smaller increases in cytokine production in response to further *in vitro* LPS or CL097 stimulations than observed for CD49a^−^ ihMφ or pbMOs.

## Discussion

The liver plays a central role in regulating immune responses and inflammation. Antigen-presenting cells in the liver, such as intrahepatic Mφ, sense and recognize pathogen-associated and danger-associated molecular patterns through pattern recognition receptors (PRRs) (34). The localization of intrahepatic Mφ within the liver sinusoids in close proximity to LSECs has been suggested to be essential for the functionality of intrahepatic Mφ. During acute or chronic liver diseases, intrahepatic Mφ have been described to control liver injury by shaping an anti-inflammatory liver immune response (35). However, due to limited availability of human liver samples, very little is known about the phenotype and function of human liver Mφ. Here we show that intrahepatic Mφ can be identified based on the expression of the integrin α1 receptor CD49a, similar to intrahepatic NK cells (11, 14), the complement component 3 receptor VSIG4 and MARCO. Our results furthermore demonstrate that liver-derived CD49a^+^ ihMφ cells exhibit an increased baseline production of cytokines and higher expression of maturation and activation markers compared to CD49a^−^ ihMφ.

Monocytes and Mφ can be sub-classified depending on the expression of cell surface markers into classical CD14^hi^CD16^lo^, intermediate CD14^int−hi^CD16^int^ and non-classical CD14^lo^CD16^hi^ cells, each exhibiting different functions (9). The CD14^lo^CD16^hi^ monocytes and Mφ subset has been associated with inflammatory responses including immune activation and antigen presentation, while CD14^hi^CD16^lo^ and CD14^int−hi^CD16^int^ monocytes and Mφ subsets preferentially exert phagocytic functions (36). Comparing peripheral blood- and liver-derived Mφ from the same study subjects, we observed that the proportion of CD14^int−hi^CD16^int^ cells in ihMφ was higher than in matched pbMOs. In contrast, CD14^hi^CD16^lo^ monocytes and Mφ subsets were present in a lower proportion within ihMφ when compared to pbMOs, confirming previous studies (10). CD14^int−hi^CD16^int^ monocytes have been suggested to possess inflammatory functions, as they are often expanded in inflammatory disorders (37). Expansion of CD14^int−hi^CD16^int^ ihMφ during fibrosis/cirrhosis has been demonstrated, indicating that this cell subset contributes to intrahepatic inflammation via its proinflammatory cytokine profile (38). As the spectrum of liver diseases in our study cohort was heterogeneous, ranging from alcoholic liver disease to HCV infection, and the size of the study cohort was small, it was not possible to determine differences between liver- and peripheral blood-derived monocytes and Mφ subsets in the context of the underlying liver diseases. Taken together, our data suggest that livers contained a higher proportion of developing transitional Mφ, which might be due to a high level of inflammation in the liver samples included in this study.

Contrary to mouse liver-derived Mφ (mouse KCs) that can be distinguished from circulating monocytes by expression of F4/80 glycoprotein, a member of the EGF-TM7 receptor family (39, 40), markers clearly distinguishing human liver-derived macrophages from monocytes are lacking. CD68 has been suggested as a possible marker for human KCs, although it is not-exclusive (41). Recently, the V-set and Ig domain-containing 4 (VSIG4), also known as CRIg, has been suggested to identify mice and human macrophages in tissues, as it is largely absent on circulating monocytes (17, 18, 22–24, 42). VSIG4 has several roles in immune regulation serving as an immunosuppressive and anti-inflammatory molecule, and was also described to efficiently phagocytose bacteria and boost elimination of intracellular microorganisms (22, 43). A recent publication furthermore suggested the presence of two differentiated monocyte/Mφ subsets in human liver samples, dependent on the expression of the PRR MARCO on the cell surface (23). Previous studies have also shown that the expression of CD49a identifies a population of tissue-resident NK and T cells in mice and humans (11–14, 27, 44–46). CD49a is the α1 subunit of α1β1 integrin, a receptor associated with adhesion of lymphocytes to collagen IV. In line with these observations for NK and T cells, we detected CD49a-expression exclusively on liver-derived Mφ, while CD49a^+^ MOs were largely absent in peripheral blood. Within ihMφ, the expression of CD69, CD80, CD83, and CD86 was higher on CD49a^+^ Mφ compared to CD49a^−^ Mφ. Our data using both flow cytometry and immunofluorescence staining of liver tissue samples furthermore showed that ihMφ consisted of a variety of subpopulations expressing different combinations of CD49a, VSIG4 and MARCO.

The liver has been traditionally described as a tolerogenic organ, with Kupffer cells (KCs) and dendritic cells representing the main cell subsets mediating tolerance. KCs suppress CD8^+^ T cell activity by several mechanisms, including overexpression of Fas-Ligand, which induces CD8^+^ T cell destruction (47); overexpression of PD-L1, which leads to cell exhaustion through PD-1 receptor on T cells (48); or secretion of IL-10, an immunosuppressive cytokine (49, 50). Although intrahepatic KCs have been shown in mice and rats to express TLRs (51, 52), those cells are hypo-responsive to endogenous LPS and also other TLR ligands (53). Nonetheless, novel antigens arising from microbial infections, such as influenza in humans, strongly activate liver macrophages (54). Predisposition for endotoxin-tolerance might be primarily due to an adjustment to PAMPs arising from intestinal microbiota, which are not posing an immunological threat. This effect might be particularly relevant during inflammation, when higher gut translocation of bacterial and viral PAMPs is observed (6). Human macrophages are classified in M1 or M2 according to their functional properties: M1 macrophages are pro-inflammatory, by secreting TNF-α and IL-12, and induce a Th1 and Th17 response, while M2 macrophages, secreting IL-10, have been shown to be anti-inflammatory and recruit a Th2 response (55). Our results are in line with endotoxin-tolerance, as CD49a^+^ ihMφ contained a higher proportion of cells expressing activation markers and higher cytokine levels at baseline, but did not strongly react to further TLR4 or TLR7/8 stimuli. Moreover, the majority of cytokine-producing cells within the CD49a^+^ ihMφ population exhibited an M1 phenotype, as they produced TNF-α and/or IL-12 at baseline. Since the production of IL-12 by monocytes/macrophages upon LPS-exposure was shown to be dependent on previous IFN-γ-mediated priming (56), our data indicate that IFN-γ-producing cells, such as intrahepatic NK or T cells, might be able to prime CD49a^+^ ihMφ.

In conclusion, we showed in this study that human intrahepatic macrophages can co-express CD49a, VSIG4 and/or MARCO. CD49a^+^ ihMφ had distinct phenotypical patterns as well as functional properties, when compared to CD49a^−^ ihMφ and peripheral blood-derived monocytes.

## Ethics Statement

This study was carried out in accordance with the recommendations of the Institutional Review Board of the medical faculty at the University of Hamburg (PV4898, PV4081, PV4780, and WF-021/11) with written informed consent from all subjects. All subjects gave written informed consent in accordance with the Declaration of Helsinki. The protocol was approved by the Institutional Review Board of the medical faculty at the University of Hamburg.

## Author Contributions

GM, HG, SL, and MA: conceived and designed the experiments. GM, HG, AEL, JK, FF, AN, and SL: performed the experiments. GM, HG, AEL, WS, LUH, AEZ, SML, KR, and TP: processed the liver samples. GM, HG, FF, JK, and SL: analyzed the data. GM and SL: performed the statistical analysis. MK, BN, and KJO: provided the clinical samples. CS, MD, and MA: provided resources. GM, SL, and MA: drafted the paper. All authors critically reviewed and approved the final version of the paper.

### Conflict of Interest Statement

The authors declare that the research was conducted in the absence of any commercial or financial relationships that could be construed as a potential conflict of interest.
