# Supplementary material for: CD49a Expression Identifies a Subset of Intrahepatic Macrophages in Humans
Source: Front Immunol. 2019 Jun 7;10:1247. doi: 10.3389/fimmu.2019.01247 (PMC6568245; doi:10.3389/fimmu.2019.01247)
Supplement: Supplementary file 1 [file Data_Sheet_1.docx]

**Supplementary Table 1. Demographic/Clinical data of individuals Figure 1**

f: female; m: male; LTX: liver transplant surgery; AKB: surgical tumor-free liver tissue resection; AST: Aspartate transaminase; ALT: Alanine aminotransferase; AP: Alkaline phosphatase; GGT: Gamma-glutamyltransferase; IRN: international normalized ratio (blood-clotting test); MELD: Model for End-Stage Liver Disease. Note: MELD Score was not determined for AKB samples.

**Supplementary Table 2. Demographic/Clinical data of individuals Figure 2/Supplementary Figures 2 and 3**

nd: non-determined; HLR: Healthy Liver Resection; Clinical values were not determined for the HLR sample.

**Supplementary Table 3. Demographic/Clinical data of individuals Figure 3**

Note: MELD Score was not determined for AKB samples. The AP and GGT levels were not determined in 1 individual.

**Supplementary Table 4. Demographic/Clinical data of individuals Figures 4/5**

Note: MELD Score was not determined in 3 individuals, the Albumin and AST levels were not determined in 1 individual.

**Supplementary Table 5. List of antibodies**

**Supplementary Code 1. Script used as a macro in Fiji to identify and quantify positive signal combinations**

run("Set Measurements...", "area mean redirect=None decimal=3");

Dialog.create("Settings");

//Dialog.addCheckbox("Binary analysis - make threshold in every channel", false);

Dialog.addCheckbox("Reference channel needs to be measured", false);

Dialog.addCheckbox("Channels are in one file", false);

Dialog.addNumber("How many channels must be measured", 2);

Dialog.addNumber("Blurryness factor for reference channel", 2);

Dialog.addString("File extension", ".tif")

Dialog.show();

//binary = Dialog.getCheckbox();

ref = Dialog.getCheckbox();

onefile = Dialog.getCheckbox();

channel = Dialog.getNumber();

sigma = Dialog.getNumber();

ext = Dialog.getString();

sigma = "sigma="+ d2s(sigma,0);

//make threshold in the reference channel to create ROIs for measurement in the other channels

if (onefile) {

dir = File.directory;

run("Split Channels");

waitForUser("Select reference channel and press OK, or cancel to exit macro");

name = getTitle();

name = substring(name, 3);

name = replace(name, ext, "_");

} else {

waitForUser("Select reference channel and press OK, or cancel to exit macro");

dir = File.directory;

name = getTitle();

name = replace(name, ext, "_");

}

if (ref) {

run("Duplicate...", " ");

}

//run("8-bit");

run("Gaussian Blur...", sigma);

run("Threshold...");

waitForUser("set the threshold and press OK, or cancel to exit macro");

run("Convert to Mask");

//run("Watershed");

run("Analyze Particles...", "clear add");

//Do measurements in the other channels

for (i=0; i<channel; i++) {

waitForUser("Select the next channel and press OK, or cancel to exit macro");

channelname = getString("Enter Channel Name. Channel name will be added to the name of the result file", "i.e. CH2");

//run("8-bit");

roiManager("Show All");

roiManager("Measure");

filename = dir+name+channelname+"-int.csv";

saveAs("Results", filename);

IJ.deleteRows(0, 20000);

run("Gaussian Blur...", sigma);

setAutoThreshold("Default dark");

run("Threshold...");

waitForUser("set the threshold and press OK, or cancel to exit macro");

run("Convert to Mask");

roiManager("Show All");

roiManager("Measure");

filename = dir+name+channelname+".csv";

saveAs("Results", filename);

IJ.deleteRows(0, 20000);

run("Close");

}

roiManager("Delete");

run("Close All");
