# Supplementary material for: CD49a Expression Identifies a Subset of Intrahepatic Macrophages in Humans
Source: Front Immunol. 2019 Jun 7;10:1247. doi: 10.3389/fimmu.2019.01247 (PMC6568245; doi:10.3389/fimmu.2019.01247)
Supplement: Supplementary file 2 [file Presentation_1.pptx]

## Slide 1
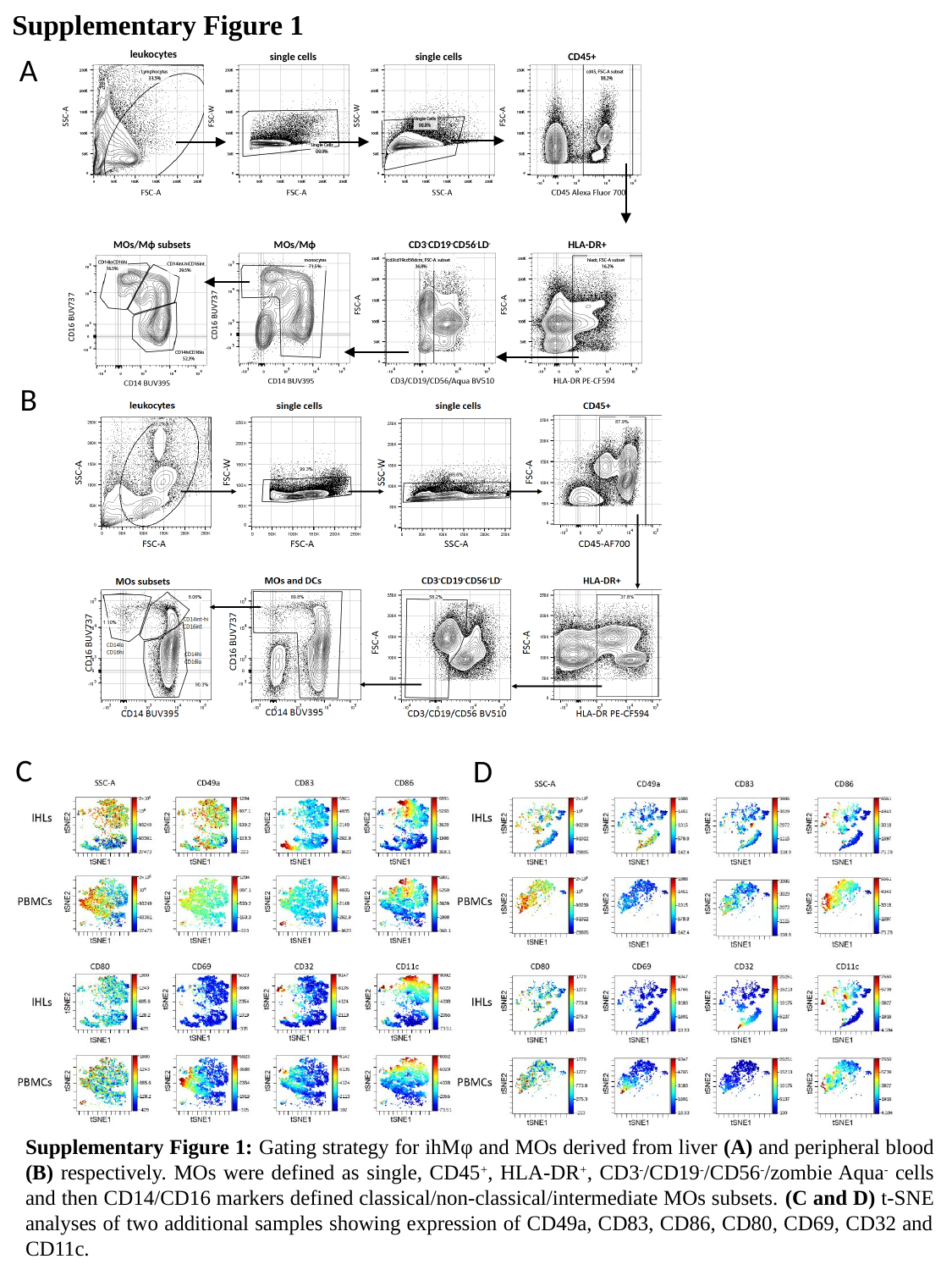

Supplementary Figure 1
leukocytes
A
single cells
single cells
CD45+
MOs/Mϕ subsets
MOs/Mϕ
CD3-CD19-CD56-LD-
HLA-DR+
B
C
D
Supplementary Figure 1: Gating strategy for ihMφ and MOs derived from liver (A) and peripheral blood (B) respectively. MOs were defined as single, CD45+, HLA-DR+, CD3-/CD19-/CD56-/zombie Aqua- cells and then CD14/CD16 markers defined classical/non-classical/intermediate MOs subsets. (C and D) t-SNE analyses of two additional samples showing expression of CD49a, CD83, CD86, CD80, CD69, CD32 and CD11c.

## Slide 2
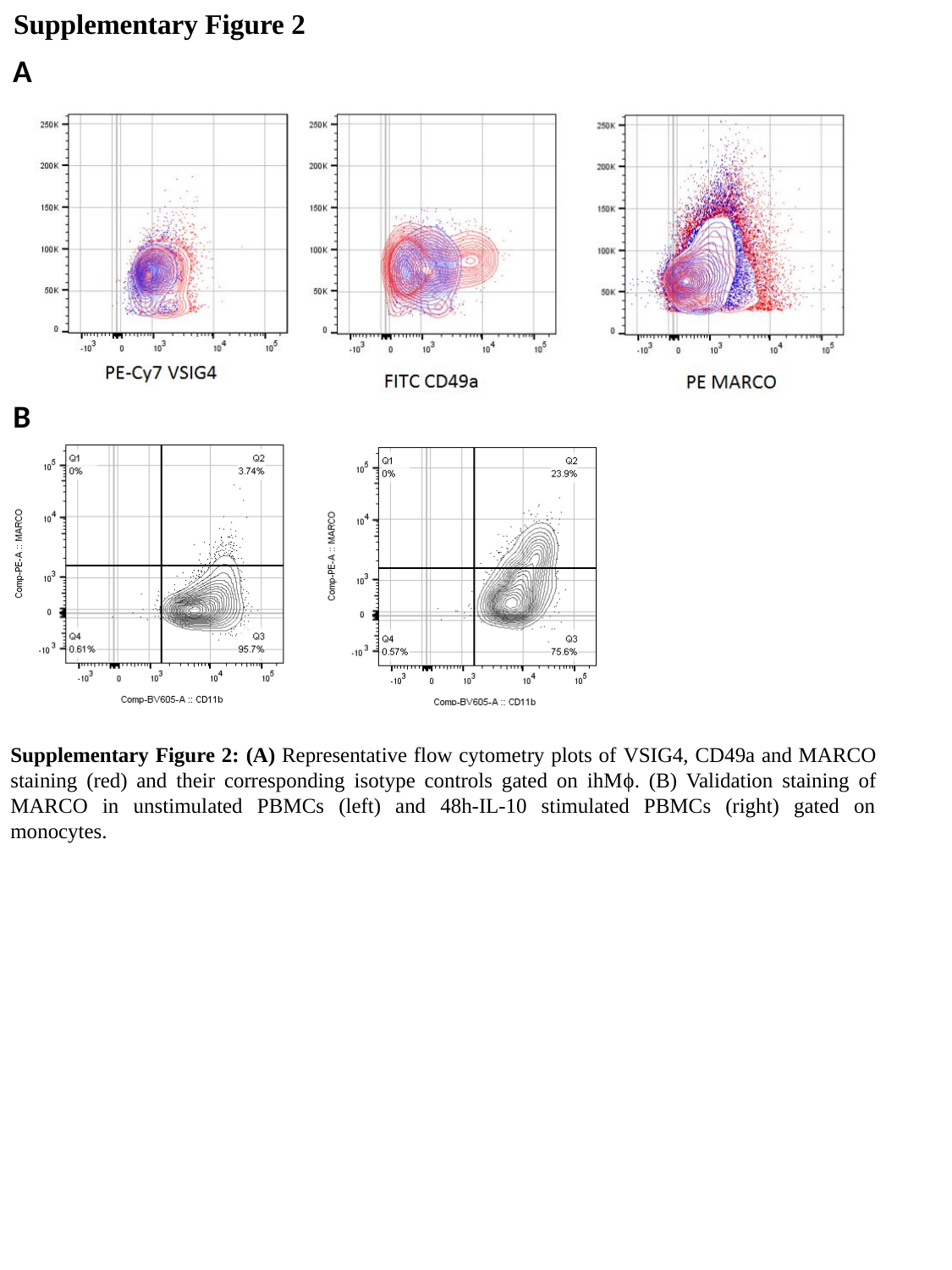

Supplementary Figure 2
A
B
Supplementary Figure 2: (A) Representative flow cytometry plots of VSIG4, CD49a and MARCO staining (red) and their corresponding isotype controls gated on ihMϕ. (B) Validation staining of MARCO in unstimulated PBMCs (left) and 48h-IL-10 stimulated PBMCs (right) gated on monocytes.

## Slide 3
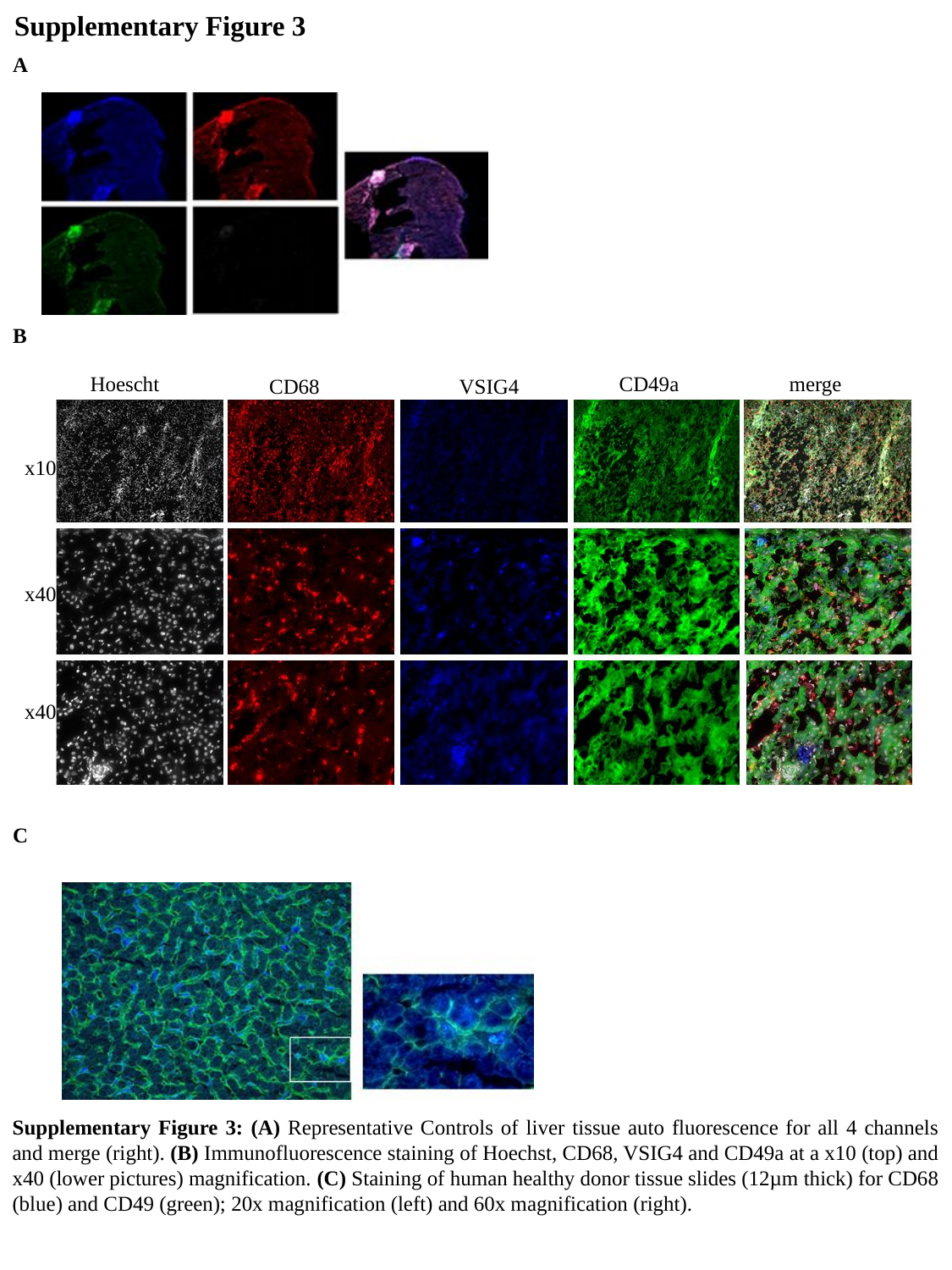

Supplementary Figure 3
A
B
Hoescht
CD49a
merge
CD68
VSIG4
VSIG4
x10
x40
x40
C
Supplementary Figure 3: (A) Representative Controls of liver tissue auto fluorescence for all 4 channels and merge (right). (B) Immunofluorescence staining of Hoechst, CD68, VSIG4 and CD49a at a x10 (top) and x40 (lower pictures) magnification. (C) Staining of human healthy donor tissue slides (12µm thick) for CD68 (blue) and CD49 (green); 20x magnification (left) and 60x magnification (right).

## Slide 4
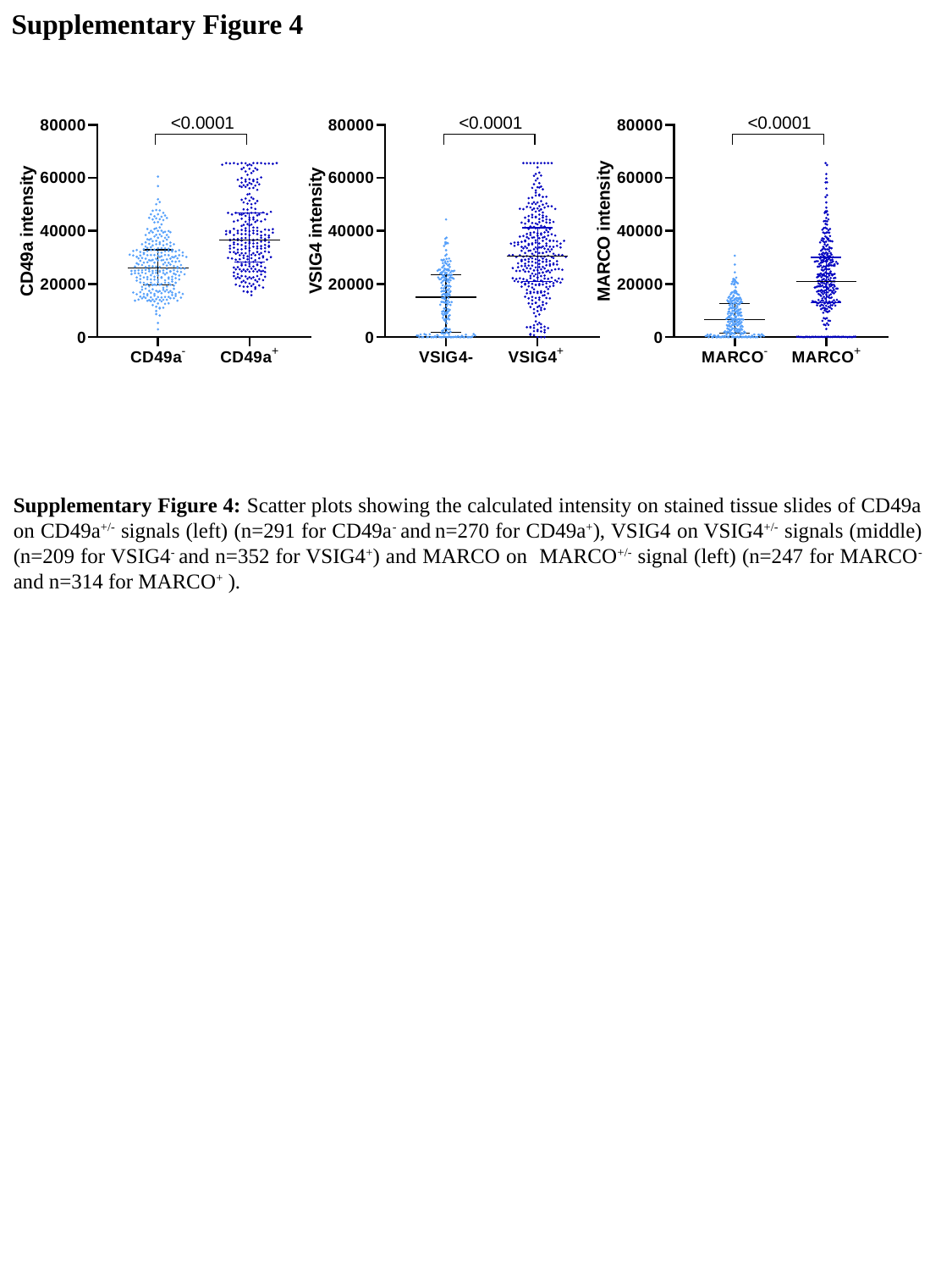

Supplementary Figure 4
Supplementary Figure 4: Scatter plots showing the calculated intensity on stained tissue slides of CD49a on CD49a+/- signals (left) (n=291 for CD49a- and n=270 for CD49a+), VSIG4 on VSIG4+/- signals (middle) (n=209 for VSIG4- and n=352 for VSIG4+) and MARCO on MARCO+/- signal (left) (n=247 for MARCO- and n=314 for MARCO+ ).
